# Anglo-American Memories1Copyright, 1910, by George W. Smalley.

**Published:** 1910-09

**Authors:** George W. Smalley


### Anglo-American Memories[1]

### Sir Thomas Barlow—Sir William Gull—Sir William Broadbent—Sir Andrew Clark

Contributed to the *New York Tribune* by George W. Smalley.

SIR THOMAS BARLOW, of whom I said something lately, has this week been elected President of the Royal College of Physicians, in succession to Sir Douglas Powell. This is the Blue Ribbon of the profession, perhaps a greater honor than a knighthood or baronetcy, though the knighthood or the baronetcy is from the King, the source and fountain of all such distinctions. But the presidency of the Royal College of Physicians is conferred by the profession itself. The Fellows of the College, who number some three hundred, are the choosing body. They vote by ballot, and the man whom they elect is the man by whom they wish to be represented before the public; the man by whom they are content to be judged. They say, in effect, of him whom they choose: "This is the Head of the Medical Profession for the time being." The public, which really and rightly has much more confidence in the judgment of the doctors upon each other than in any lay reputation, accepts that. When you say of a physician, "He is a doctor's doctor," you have said about all you can.

The President of the Royal College of Physicians has, no doubt, duties which are not medical. He has executive, administrative, consultative duties; and the very important duty of dining with the Lord Mayor, the Corporation of the City of London and the City Companies. In discharging these latter functions he incurs, I suppose, less risk than most men incur. But, risk or no risk, these feasts have to be faced. Between all Corporations, Guilds and Colleges there is a kind of freemasonry. They have points of contact, of sympathy, and are likely to stand by each other in difficulties. Whether dinners were invented as a test and standard of friendship, I cannot say. But go to which of them you like, you will find a collection of the Heads of other Companies, Colleges, and the like, not all, perhaps, dinner giving, but all willing victims of others' hospitality.

The Royal College of Physicians is also a Senate or Parliament; with powers of legislation and of professional guidance and discipline. The Fellows of this College are Trustees for the whole Profession. The President has an authority of his own, depending in part on statutes and on custom, in part on his personal authority. In the latter Sir Thomas Barlow will not be found wanting. It is not the less, it is perhaps the greater, for the genial good nature which accompanies it. I said to him once:

1. Copyright, 1910, by George W. Smalley.

"Sir Thomas, you have one quality which must be a great drawback to your success."

"Dear me, what is that?"

"When you come into a room, your patient at once thinks himself better, and even doubts whether he need have sent for you at all, and so gets well much quicker than he ought. It's taking money out of your pocket."

"Very good. I'll take care *you* don't get well too soon."

There was an electioneering story—oh, no politics in it—the other day with an equally serious, but not more serious, side to it. Men were discussing the system of plural voting still prevailing in this country, and certain to prevail so long as votes, or any votes, are based on a property qualification. Said a well-known doctor:

"I have sixteen votes, all of which I am going to poll."

"But how?"

"Oh, I have two votes of my own and I have fourteen patients who are of the wrong party and not one of them will be well enough to go out till after election."

Think how completely non-political must be a profession of which an eminent member can tell a story like that and run no risk of being misunderstood. The traditions of honor are indeed high among English doctors, nor could they be in better keeping than now in Sir Thomas Barlow's.

One of his predecessors, Sir William Gull, was also not merely fashionable and popular, but recognised by his associates as a scientific practitioner. Sir William Jenner was perhaps reckoned by the medical profession the best all-round man ever known. Sir William Gull was not far off, yet there is an anecdote of him which suggests that he put a very high value on the average capacity of doctors. He was asked to go a long distance into the country to see a patient. He declined. He was told that any fee he liked to name would be gladly paid. Still he declined, saying there were cases he could not leave, and when he was pressed further the great man burst out:

"But why do you want me? There are five hundred doctors in London just as good as I am."

Which, perhaps, was not quite true.

Sir William Broadbent said almost the same thing to me, twenty years ago, and more, when I asked him to see Mr. Hay, whom I had just left in his rooms, in Ryder street, St. James's, to all appearance extremely ill. Hay said in his emotional way:

"Broadbent is the only doctor I believe in. If you don't bring Broadbent bring nobody. Let me die."

But Broadbent said no. He was starting to catch a train for a life and death consultation in the country. He must not miss his train.

"But there's time enough. See Hay on your way to the train. Give him five minutes, and let somebody else do the rest."

"I shall let somebody else do the whole."

"Hay will see nobody unless he sees you first."

"There are plenty of men as good as I am. I will give you half a dozen names."

"I want none of them. I want you. You know you can stop your carriage for five minutes as you drive to the station."

"My carriage has not come round."

"My hansom is at the door. Drive with me and let your carriage follow."

Finally he did. When he came out of Hay's bedroom he was a very angry man. He said:

"Your friend has a bad attack of indigestion. He will be all right in an hour."

And away he went. An angry man is not always a just man. Hay—God bless his memory—thought himself suffering from a heart attack. There is, I believe, a medical analogy between the symptoms of heart disease and violent indigestion. I had left him on the floor, almost in convulsions. How was he to know it was not heart disease, to which he believed himself subject? Hay was not then, to the English, so great a man as he afterward became. He had not been Ambassador, nor Secretary of State, nor dictated to the European Powers a new policy in the East. I ought not to use the word dictated. It is not descriptive of Hay's methods, which were persuasive. Nor does one Power dictate to another. Let us say he had secured by the adroit use of accepted diplomatic methods the adhesion of the European Powers to his proposals in respect of China. No American Secretary of State had ever made so original or beneficent a use of his power. He had brought his country once for all into the great world partnership of great Powers the world over.

Sir William Broadbent did not foresee that. He could not. If he had, he might have been less angry, for he was thought to be considerate of greatness in all its forms, or in many of them. He liked patients of distinction, which is no reproach. He had many of them. But the odd thing was that he seemed never quite able to overcome his awe of rank and title. In a company of persons of rank his manner was not that of an equal. He used to address persons of rank as a servant addresses them; or it might be kinder to say as inferiors in position used to address their superiors two or three generations ago. And always with embarrassment.

Another celebrated man of medicine, Sir Andrew Clark, had an almost factitious renown as Mr. Gladstone's doctor, and Mr. Gladstone was a very good patient, in one sense. One thing this famous physician had: he had absolute confidence in himself. Or, if no doctor has that, he had enough to give his patient confidence, which is perhaps not less important. Old Abernethy used to say: "The second best remedy is best if the patient thinks it best." And I suppose that is true of doctors as of remedies If Sir Andrew doubted, he never allowed you to see that he doubted. Like all these great men, he had a social as well as medical popularity, and he was very good company at dinner and after.

One evening I met him at a pleasant house where there was a good cook and the company, including the host, did not exceed six—all men. We all noticed that Sir Andrew drank champagne. Presently one of the men said:

"You don't allow us champagne, Sir Andrew, but you allow it to yourself."

"Oh, I have had a long day, and I am very tired, and I must have it. Besides, when I get home there'll be thirty or forty letters to answer."

So the champagne flowed on, like the water, as Mr. Evarts said, at one of President Hayes's White House dinners. Sir Andrew drank no more than anybody else. It was only because of his habit of prohibiting it to others that we noticed whether his glass was full or empty. As we went upstairs I said to him:

"Do you mean that after all that champagne you are going to answer thirty or forty letters when you get home?"

"No, certainly not."

"Then, what did you mean?"

"What I meant was that after my champagne I should not care whether they were answered or not."

It was Sir Andrew Clark who said of Mr. Gladstone, some fifteen years before his death at eighty-eight, that there was no physiological reason why he should not live to be 120. If that was meant as a prophecy it had the fate of most prophecies.

London, March 25, 1910.

---

**The Limitation of the Caloric Method of Infant Feeding**

Henry Dwight Chapin of New York, *Medical Record,* May 28, 1910, states that caloric feeding of infants is a failure, on account of the absence of any index of the amounts of protein needed for the infant's growth. If only as much heat-producing material is taken in as there is heat given off, there can be no storage of protein for growth. The amount of food needed for growth cannot be determined by the amount of heat excreted. Foods that are of equal heat-producing value, and equally digestible are not interchangeable for different individuals.

